# Collapse of Lung as Described by West

**Published:** 1855-03

**Authors:** 


### ART. IV.— *Collapse of Lung as described by West.*

It has long been known that infants of feeble nervous energies, not unfrequently suffered from imperfect expansion of the lung at birth, a condition sometimes continued for many weeks of a wretched existence, and, finally, terminating in death.

Cases also occur in infants who have previously breathed well, of an analagous nature. Occasionally cases were met where cough was unattended by fever, but which presented dullness on percussion, and either bronchial respiration or impaired and feeble vesicular murmur, thus offering some of the physical signs of pneumonia, while the rational symptoms were wanting. On *post-mortem* examination, these lungs were found solidified, and it was supposed to be an irregular and masked form of pneumonia. Many of these case presented irregular mottled patches over the lung, resembling lobular pneumonia, and mistaken for it. Louis made a distinction in the *post-mortem* appearances, and gave the name of "carnification" to the appearance alluded to. At the same time it is evident that he did not recognize the true causes and nature of the appearances noted. For the first intelligent description of them we are indebted to West.

The pathological appearances of the lung are briefly these: Patches of solidified lung are scattered throughout its substance in variable proportion, presenting to the eye the appearance of lobular pneumonia. Sometimes these scattered patches involve the entire substance, sometimes they exist as indentations on its surface. When general, the size of the organ is diminished. The important pathological difference between it and pneumonia (hepatization) is, that the lung may be readily inflated by the blow pipe, and then resumes its normal specific gravity and provosity.

The causes of collapse of lung are as numerous as the causes of nervous exhaustion. Any thing which may so affect the nervous energies as to impair the *besoin de respiré*, or so diminish the physical strength as to render the patient incapable of fully expanding the lung, may act as a cause. Thus it supervenes on exhausting diarrhœas, bronchitis, or pertussis. And it may doubtless follow deficient action of the pneumo-gastric nerve.

The symptoms will accordingly vary considerably in different cases. The rational signs are extremely uncertain, and the nature of the disease may, therefore, easily pass undetected, while the physical signs are almost unerring, and cannot fail to lead to an understanding of the case.

A few cases will best illustrate the phenomena and treatment of collapse of lung.

Case I. March 31, 1852. Called to child of E. T., aged fifteen months. The patient had just been abandoned by the attending physician as having incurable disease of the heart.

*Rational Signs.* Emaciation; dry, cool skin; complexion livid; thirst; great restlessness, with a feeble, forceless cry; respiration very rapid; pulse about 140, varying in force; cough, without much expectoration; mind clear.

*Physical Signs. Percussion.* Flatness over right chest; dullness over left.

*Auscultation.* A very rapid and tumultuous action of the heart obscured the pulmonary sounds. The respiratory murmur was nevertheless more distinct on the left than on the right side.

*Aspect.* Breathing abnormal; impulse of the heart visible, with a quick sharp, jerking motion. Right side does not expand as much as left, and neither of them expand normally.

The patient had had a cough for sometime, had "taken cold" repeatedly and had gradually lost strength and flesh.

The treatment was stimulant and tonic. Brandy, quinine, ammonia, and iodide of potassium were given internally, and a stimulating liniment applied to the chest. The child improved slightly for a time, and the case offered some chances of recovery for a fortnight, when hydrocephalic symptoms appeared, and death followed.

*Post-mortem.* Only the cavity of the chest was opened. The characteristic pathological signs of collapse of lung were found. The whole right lung was involved, and a large portion of the left. The heart was natural in appearance, with the exception of unusually large fibrinous clôts extending from its cavity into the pulmonary vessels.

Case II. Male child of N. C., aged 3 years. This little boy had had, during the preceding spring, a "hard cold," followed by fretfulness, emaciation, cough, expectoration, and gradually increasing debility. He was supposed to have phthisis, and had been abandoned as incurable.

*Rational Signs.* Debility, unable to walk; fretful, with an anxious expression of countenance; variable appetite; cough, with variable amounts of expectoration.

*Physical Signs.* Dullness on percussion over the base of right lung, and more or less impaired resonance in all parts of the chest. Bronchial respiration with mucous râles. Voice hoarse; impulse of cough feeble.

*Aspect.* Abdominal respiration, and deficient expansion of chest, especially on the right side.

The treatment was stimulant, tonic, and expectorant. Wine whey, animal food, warm clothing, out-door air, iodide of potassium, with some stimulating expectorants were given, with frequent modifications to meet the daily exigencies of the case. The improvement was uniform and steady, and after a month of treatment a very good degree of health was obtained. Since that time his health has continued good.

Case III. Female child of J. N. G., aged 16 months. I saw this child, in consultation with Dr. Richards, of Medina, Orleans Co. She had been for a few days previously under homœopathic treatment. Four months previously she had had pertussis severely, and had never recovered from the cough. She had grown none during this period, and was thin and emaciated. Up to a few days previously she had been bright and playful. Her gums were then swollen and painful, and were lanced. She seemed also to take cold. At this time she became stupid, did not speak, though capable of being roused, and lay in a quiet but semi-comatose condition. The diagnosis of the homœopath was hydrocephalus.

*Rational Signs.* The rational signs at the period of consultation were, an extremely sleepy or stupid condition, is roused with some difficulty; head cool; pupils of eyes sensitive to light; skin moderately warm, slightly livid; pulse about 100, with an occasional inequality in the force of the beat, and a slight irregularity in the rhythm; respiration at times scarcely perceptible, attended by a few labored inspirations occurring at intervals. The whole appearance was that of a child suffering from an overdose of opium. The cough was dry, feeble, forceless.

It will be noticed that the rational signs point with considerable meaning to hydrocephalus. Indeed it was necessary to give due weight to negative symptoms to make a different diagnosis. Thus the head was cool; the pupils sensitive; the position of the thumbs natural; there was no restlessness moaning, or rolling the head, and no disposition to vomit. The countenance was placid.

*Physical Signs.* The physical signs were as unmistakeable as they were unindicated by the general symptoms. There was great dullness over the right lung on percussion. The left lung was clear. On the right side the respiratory murmur was scarcely perceptible, neither was there much bronchial respiration. On the left side the respiration was exaggerated. There was abdominal breathing, and imperfect expansion of the right side.

The treatment comprised an expectorant of syrup ipecac, spts. nitre dulc., and spts. ammon. aromat., in the hope of encouraging secretion, with iodide of potassium, given without any special theory, except that it has uniformly seemed to act favorably as a tonic and alterative in these cases. Beef tea was given liberally, with an occasional warm saline bath, and a stimulating liniment applied to the chest.

Two days after I saw her again. She seemed to have lost ground. The hydrocephaloid symptoms, though no worse, were persistent. Pulse was more regular, and the rhythm of the respiration improved. There was more lividity of the surface. The right lung was flat on percussion over its whole extent, with an absence of respiratory sounds. The right side was also motionless, and diminished in size. Breathing abnormal. Bowels soft. She seemed to have lost the *besoin de respiré*, and to breathe like an animal with the *par vagum* severed.

The treatment was changed. Mustard paste was ordered to the chest, beef tea to be given liberally, gr. ss. of quinine every three hours with a little brandy, and acetate of ammonia in the hours of the interval.

The prognosis was doubtful. Should the system fail to accommodate itself to this cyamosed condition the stupor would become merged in profound coma; head symptoms and death would follow. On the other hand the case was now as bad as it could be unless the left lung should become involved. The general symptoms were not worse in any marked degree than before, and there was, therefore, a hope that could the system be supported nervous energy would be acquired, and the lung again slowly expand.

The notes of this case have thus far been given without any further information of its results. Nothing can illustrate more forcibly the high value of physical exploration than a case like this, where the head symptoms have assumed such a prominence as to easily confound the diagnosis. It is evident that had a correct diagnosis been made at the time she was placed under homœopathic management, a supporting treatment would have found a far greater chance for success in a nervous system still retaining some energy, and not yet overpowered by the sedative influences of cyanosis.

P. S. This case terminated fatally three days after the last visit. The pulse, during this interval, improved; the breathing was not more embarrassed, but the coma continued, and finally a spasm occurred — rigidity rather than convulsion — lasting for several hours. The breathing was stridulous (croupy as described by the parents.) A few minutes after the relaxation of the spasm, death occurred. The countenance remained placid throughout. The stridulous breathing was probably dependent on partial or complete paralysis of the par vagum of the right side. H.

